# Impact of whole-genome amplification on the reliability of pre-transfer cattle embryo breeding value estimates

**DOI:** 10.1186/1471-2164-15-889

**Published:** 2014-10-12

**Authors:** Habib A Shojaei Saadi, Christian Vigneault, Mehdi Sargolzaei, Dominic Gagné, Éric Fournier, Béatrice de Montera, Jacques Chesnais, Patrick Blondin, Claude Robert

**Affiliations:** Laboratory of Functional Genomics of Early Embryonic Development, Institut des nutraceutiques et des aliments fonctionnels, Faculté des sciences de l’agriculture et de l’alimentation, Pavillon des services, Université Laval, Québec, G1V 0A6 Canada; L’Alliance Boviteq Inc, 19320 Grand rang St-François, Saint-Hyacinthe, J2T 5H1 Québec Canada; L’Alliance Semex Inc, 130 Stone Road West, Guelph, N1G 3Z2 Ontario Canada

**Keywords:** Bovine early embryo, Embryo biopsy, Whole-genome amplification, Genotyping, Genomic breeding value, Genotype imputation, Pre-Implantation genetic diagnosis, Multiple displacement amplification

## Abstract

**Background:**

Genome-wide profiling of single-nucleotide polymorphisms is receiving increasing attention as a method of pre-implantation genetic diagnosis in humans and of commercial genotyping of pre-transfer embryos in cattle. However, the very small quantity of genomic DNA in biopsy material from early embryos poses daunting technical challenges. A reliable whole-genome amplification (WGA) procedure would greatly facilitate the procedure.

**Results:**

Several PCR-based and non-PCR based WGA technologies, namely multiple displacement amplification, quasi-random primed library synthesis followed by PCR, ligation-mediated PCR, and single-primer isothermal amplification were tested in combination with different DNA extractions protocols for various quantities of genomic DNA inputs. The efficiency of each method was evaluated by comparing the genotypes obtained from 15 cultured cells (representative of an embryonic biopsy) to unamplified reference gDNA. The gDNA input, gDNA extraction method and amplification technology were all found to be critical for successful genome-wide genotyping. The selected WGA platform was then tested on embryo biopsies (n = 226), comparing their results to that of biopsies collected after birth. Although WGA inevitably leads to a random loss of information and to the introduction of erroneous genotypes, following genomic imputation the resulting genetic index of both sources of DNA were highly correlated (r = 0.99, P<0.001).

**Conclusion:**

It is possible to generate high-quality DNA in sufficient quantities for successful genome-wide genotyping starting from an early embryo biopsy. However, imputation from parental and population genotypes is a requirement for completing and correcting genotypic data. Judicious selection of the WGA platform, careful handling of the samples and genomic imputation together, make it possible to perform extremely reliable genomic evaluations for pre-transfer embryos.

**Electronic supplementary material:**

The online version of this article (doi:10.1186/1471-2164-15-889) contains supplementary material, which is available to authorized users.

## Background

Pre-implantation genetic diagnosis (PGD) is used in human fertility clinics, mainly to detect genetic disease factors that may have been passed on to the embryo [1–4]. Such diagnostics can be carried out using polar bodies or embryonic cells collected by biopsy [5–9]. Extraction of the genome from polar bodies is less invasive than a blastomere biopsy but yields poorer quality gDNA and ignores the effects of the paternal genome, leading to less precise information [10–14]. In livestock, embryonic genotyping is generally performed on trophoblast cells at the blastocyst stage and is used primarily for sex determination. This procedure is performed routinely, on-site and involves the detection of a Y-chromosome-specific sequence by different means of DNA amplification [15–19]. In addition to sex determination, embryonic cell sampling and DNA amplification may be used to determine genotypes at specific loci.

The development of high-throughput platforms capable of genotyping up to ten thousand [20, 21], tens of thousands [22–24] and even several hundred thousand [25, 26] of single-nucleotide polymorphisms has led to the development of whole-genome-based selection of livestock [15, 27, 28]. Currently, an emerging trend in the dairy cattle industry involves determining genetic merit at birth using breeding values from genotypic information provided by these high-throughput genomic platforms. For the purposes of further increasing selection pressure and reducing generation interval, there is an increasing demand in determining breeding values readily on early (pre-hatching) embryo genomics before embryos are transferred into recipients. By doing so, it is possible to identify high-genetic-merit individuals before transfer and thus decreasing the number of embryo transfers which represents considerable economic value [29]. However, the challenges arising from genotyping a handful of candidate loci are exacerbated when large numbers of loci are distributed across the entire genome.

Because an embryo biopsy typically contains about 10 to 15 cells, whole-genome amplification (WGA) is necessary in order for enough material to be available for genotyping. Current WGA methods involve one of two approaches: PCR-based (thermo cycling) and non-PCR-based (isothermal amplification) [30, 31]. Several PCR-based WGA methods have been developed, such as primer extension pre-amplification (PEP), degenerate oligonucleotide-primed PCR (DOP-PCR), tagged random primers (T-PCR) and ligation-mediated amplification (LMA) PCR. These vary in efficiency, coverage and range of applications and have different limitations such as amplification bias, generation of relatively short products (<3 kb), which may not be suitable for downstream applications, incomplete genomic coverage and a high possibility of randomly introducing point mutations into the products [32, 33]. The most recent PCR-based WGA technology for single-cell amplification is quasi-random primed library synthesis followed by PCR amplification (QPLS-PCR). This approach has been used to support human *in vitro* fertilization by providing pre-implantation genetic screening for aneuploidy and genetic testing for familial single-gene disorders. Studies have shown that QPLS-PCR technology overcomes limitations generally associated with PCR-based WGA and can be applied successfully to very limited genomic DNA samples such as embryo blastomeres or oocytes [34–36]. Among the non-PCR based methods, multiple displacement amplification (MDA) protocols are the most commonly used and have been developed for non-specific DNA expansion. This isothermal reaction uses random primers (exonuclease-resistant hexamers) to initiate DNA replication by a bacteriophage DNA polymerase such as the Phi29 enzyme, which exhibits strong DNA displacement capabilities [37]. Low error rate and low amplification bias, consistent DNA amplification and longer products (>10 kb) than obtained using PCR-based WGA approaches are the main advantages of the MDA. However, MDA is more sensitive to DNA quality and quantity as well as stochastic effects, leading to reduced genome coverage which in turn results in missing genotypes and allele dropout at heterozygous loci [38, 39]. Another technology based on single-primer isothermal amplification (SPIA) has been introduced, comprising a linear DNA amplification process that uses a DNA/RNA chimeric primer containing the tag sequence to initiate DNA polymerization which is followed by cycles of primer replacement through the removal of the RNA portion of the SPIA primer using RNase H.

Considering the diversity of available WGA technologies, the current challenge is thus to identify a WGA technology that reliably amplifies entire mammalian genomes, starting from a biopsy containing 15 or fewer embryonic cells, that is, less than 100 pg of genomic DNA. The objectives of this study were therefore: (i) to compare the performance of MDA, QPLS-PCR, LMA and SPIA in whole-genome amplification using samples of standardized source and size, (ii) to evaluate the fidelity of the selected methodology by comparing whole-genome genotypic data obtained from an embryo biopsy to unamplified DNA collected post-natally from the corresponding calves, and (iii) to use the WGA-derived genotypic data to generate accurate evaluations of the genetic merit of pre-transfer embryos.

## Results

### Sample production

In order to compare different whole-genome gDNA extraction and amplification technologies using standardized samples, a bovine fetal fibroblast primary culture was set up as the sole source of cells. Female Holstein fetal tissue was selected because of the importance of female embryo selection in the commercial context and of the predominance (95%) of the Holstein breed in the Canadian dairy herd [40]. Four sample sizes were examined: (i) 1.5 μg of gDNA for unamplified reference genotypes; (ii) approximately 420 ng of gDNA from 70,000 cells for testing the genomic DNA extraction systems on large samples; (iii) 10 ng of gDNA for testing the systems using the manufacturers’ recommended input, (referred to as high gDNA input); (iv) and the quantity of gDNA extractable from 15 cells to represent an embryo biopsy (referred to as low gDNA input).

### Identification of the most efficient genomic DNA extraction methods

**Figure 1:**
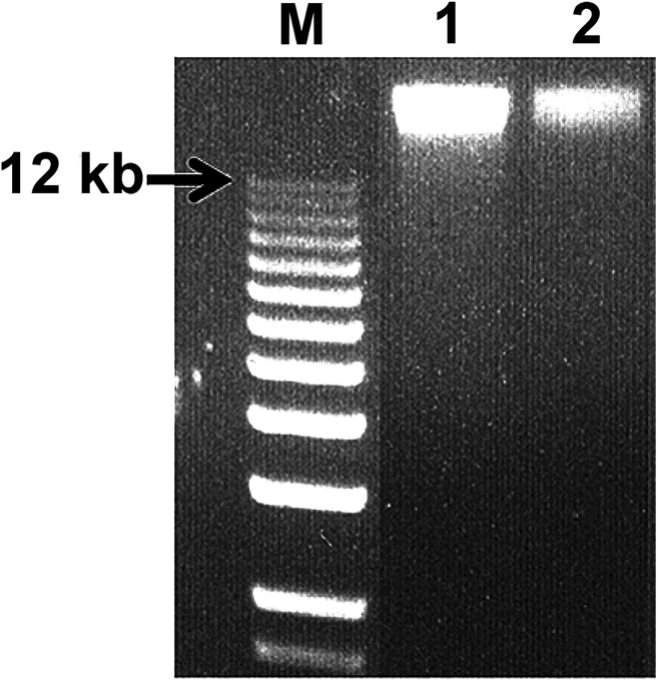
**Fragment size of genomic DNA extracted from different gDNA extraction kits.** M: DNA molecular size marker. Lane 1: ChargeSwitch gDNA Micro Tissue kit; Lane 2: Quick gDNA MicroPrep kit. The cropped image is not representative of genomic DNA yield, since end volumes differed.

To identify the most efficient genomic DNA extraction method, four commercial kits and two homemade methods were examined using samples containing about 70,000 cells. Recovery was estimated at 42–62% for DNeasy Blood & Tissue Kit, 53–63% for QIAamp DNA mini kit, 72–83% for ChargeSwitch gDNA Micro Tissue kit, 35–40% for the proteinase K treatment, and 33–46% for proteinase K followed by phenol extraction. Since gDNA integrity strongly affects WGA quality, the extracted samples were run on agarose gels to estimate the extent of gDNA fragmentation, as illustrated by DNA smear. Our results showed that the ChargeSwitch gDNA Micro Tissue kit system yielded the most intact gDNA (data not shown). It was identified as the best overall for the recovery of large gDNA input and was used as the reference gDNA extraction procedure. Therefore, except for the Ovation WGA System, the ChargeSwitch gDNA Micro Tissue kit system was used for all of the WGA technologies with high (10 ng) gDNA input. In the case of low (15 cells) gDNA input, the ChargeSwitch gDNA Micro Tissue kit was only used with the LMA-based WGA method, since the MDA-based and QPLS-PCR-based WGA methods each have their own built-in gDNA extraction technique. For the SPIA-based WGA method, Quick gDNA MicroPrep kit was used and exhibited fairly satisfactory gDNA extraction in comparison with the ChargeSwitch gDNA Micro Tissue kit (Figure 1). Therefore at comparable gDNA quality and quantity, differences in the output from the amplification reactions are attributable solely to the WGA systems.

### Impact of gDNA input on the performance of different WGA technologies

**Figure 2:**
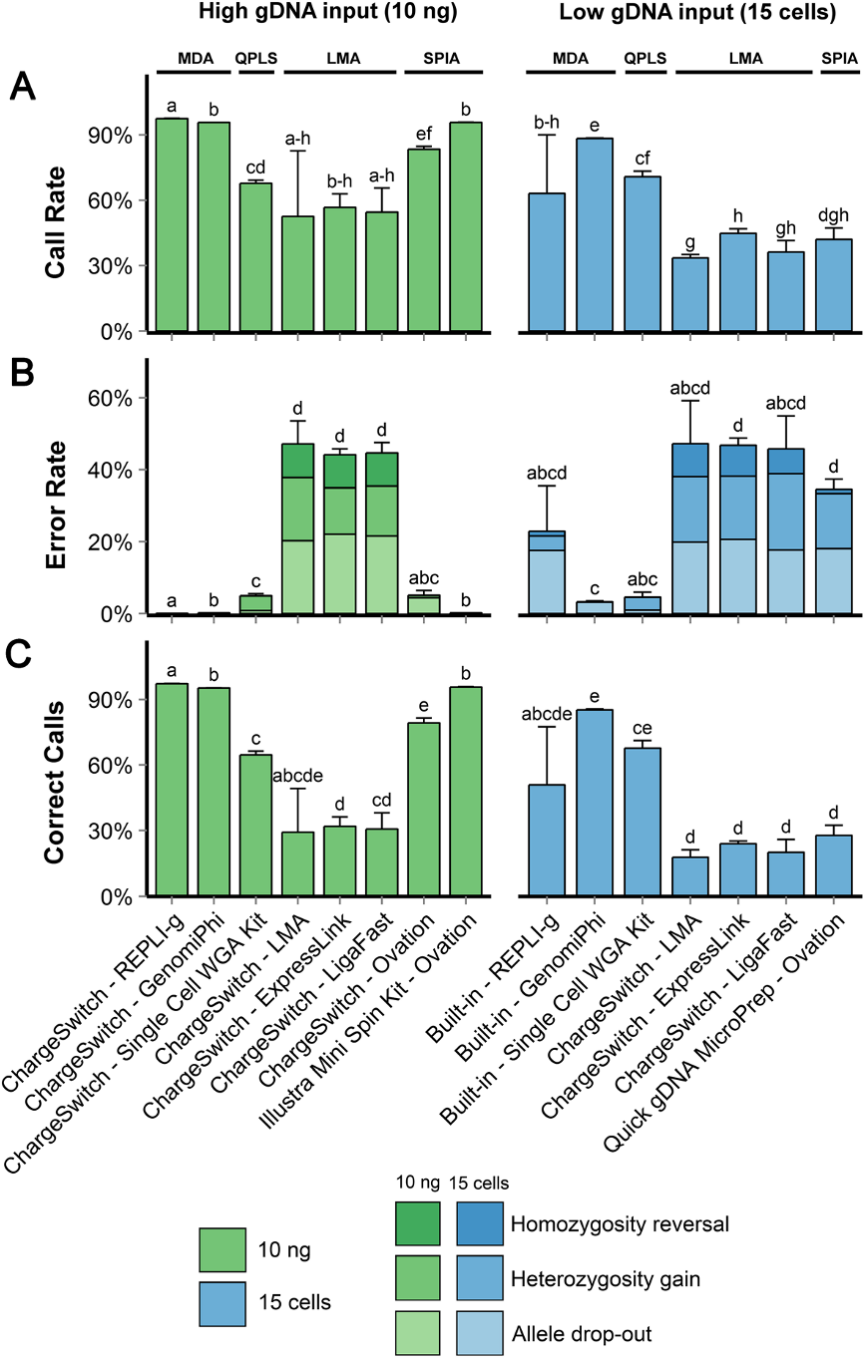
**Performance metrics for all tested WGA methods. A)** Call rate, the proportion of loci where a call was provided, correct or not. **B)** Error rate, split into the three possible categories of errors: allele drop-out (AB- > AA), heterozygosity gain (AA- > AB) and homozygous reversal (AA- > BB). The error rate is calculated relative to the number of calls provided by the method. Here error bars represent the standard deviation of the overall error rate, and not of individual types of errors. **C)** Proportion of correct calls, or loci where the method provided the correct genotype. A “no call” where the reference provided a genotype is considered a failure to provide the correct call. Error bars represent one standard deviation. Bars with different letters represent data that are significantly different (p<0.05), as determined by Post-hoc analyses using Games-Howell test. LMA: Ligation-Mediated Amplification; MDA: Multiple Displacement Amplification; QPLS: Quasi-random Primed Library Synthesis followed by PCR amplification; SPIA: Single Primer Isothermal Amplification.

To assess which approach performed best, different performance metrics were calculated for all WGA methods on both high (10 ng) and low (15 cells) gDNA inputs (Figure 2). To be considered suitable for embryonic genotyping, the methods must not only provide as much information as possible, but do so reliably and robustly. Therefore, genomic coverage as well as overall error rate and extent of variability between technical replicates were considered.

– ***Impact on call rate***

The call rate of a method is the proportion of loci where the method provided a genotyping call, regardless of its correctness. For high gDNA input, MDA-based WGA technology performed better or equally well to SPIA-based WGA methodologies, which in turn performed better than QPLS-based WGA technology (Figure 2A). The LMA-based WGA methods exhibited the lowest call rates with very high variability. With low gDNA input, most methods generated notably less genotyping call rates than they did with high gDNA input, with the exception of the QPLS-based WGA system. The MDA-based (REPLI-g Mini kit) and the SPIA-based (Ovation WGA System) WGA methods were the most impacted by the reduction in gDNA input. For the latter, given the different gDNA extraction methods used for each types of gDNA inputs, the drop in performance for the SPIA-based WGA systems could also be explained by a DNA incompatibility brought by the extraction process. Overall, the MDA-based WGA Illustra GenomiPhi V2 DNA amplification kit offered a better genomic coverage with the low gDNA input (15 cells) than the QPLS-based WGA, which in turn performed better than both the SPIA- and LMA-based WGA systems (Figure 2A).

– ***Impact on the overall error rate***

Error rates represent the proportion of incorrect calls including allele drop-out events (loss of heterozygosity) as well as allele drop-ins (gain of heterozygosity and homozygosity reversal). These errors arise mainly from imbalances in template representation and from random introduction of point mutations caused by copying errors. The loss of genomic information only becomes apparent if the original genotype was heterozygous whereas copying errors can create new alleles that were absent in the original sample.

Overall, error rates followed the same trends as call rates. With the manufacturers’ recommended minimal gDNA input (10 ng), MDA-based WGA technologies performed better or equally well than the SPIA-based WGA systems, followed by QPLS-based WGA. All of the LMA-based WGA methods trailed far behind, with error rates of about 40%. It is interesting that the error rates for the two methods (ChargeSwitch gDNA Micro Tissue and Illustra Tissue and Cells genomicPrep Mini Spin Kits; magnetic bead-based and column-based, respectively) used to extract gDNA for the SPIA-based WGA (with 10 ng gDNA input) varied widely (5.1 ± 1.2% vs 0.18 ± 0.02%) clearly showing the impact of the gDNA extraction step on WGA performance.

At low gDNA input, the MDA-based WGA Illustra GenomiPhi V2 DNA amplification kit and the QPLS-based WGA Single Cell WGA Kit performed very similarly, though the former exhibited an abundance of allele drop-out, while the latter suffered mostly from heterozygosity gains (Figure 2B). Both kits still performed much better than the REPLI-g Mini kit (MDA-based WGA) or SPIA-based WGA, whose error rates increased significantly with the reduction of gDNA input. All of the LMA-based WGA methods had an equally poor performance with the 15 cells gDNA input as it did with the 10 ng gDNA input. One particular LMA-based WGA method, with an overall error rate of 61%, had almost the same overall error rate as would be expected from randomly generated genotypes (66%) (Figure 2B).

A very rare type of error is homozygous reversal, which means a shift from one homozygous genotype to another (*e.g.* AA → BB). Although the frequency of this event was low (<1%) for MDA-, QPLS- and SPIA-based WGA methods (both high and low gDNA inputs), it was relatively high for the LMA-based WGA methods with ranges of 6 ~ 11 for high gDNA input (10 ng), 3 ~ 20 for low gDNA input (15 cells), and averages of 7 ~ 9 for all methods (Figure 2B). This was unexpected, since the mechanism by which it occurs requires both dropout and drop-in of alleles.

– ***Impact on the proportion of correct calls***

The call rate is the most straightforward metric because it is directly measured by the genotyping platform while error rates are more discrete since they can only be detected if the correct genotypes are known. Therefore, focus was given to identify the platforms that yielded the best absolute proportion of correct genotyping calls. To estimate genetic merit, a lower genomic coverage could be compensated by a low error rate and vice versa since in practice, genotyping errors are prone to escape correction since the true genotypes are unknown. At 10 ng DNA input, the same performance order as for genomic coverage (call rates) can be observed, with MDA- > SPIA- > QPLS- > LMA-based WGA technologies (Figure 2C). With the gDNA input of 15 cells, the MDA-based WGA Illustra GenomiPhi V2 DNA amplification kit showed the best performance. Although its mean is not significantly different from the REPLI-g Mini and Single Cell WGA kits, its much lower variance makes it the most attractive candidate for commercial embryo genotyping. The QPLS-based WGA performed better than all of the SPIA- and LMA-based WGA methods, which consistently generated a lower number of correct calls (Figure 2C).

– ***Locus-specific biases on the call rates and error rates***

As expected, the variance among unamplified samples was very low with 97.7% of the potential targets generating a positive signal (erroneous or not) for all three replicates (Tables 1 and 2). Moreover, 99.6% of all potential targets generated a positive signal on at least one of the three reference replicates. We believe that the remaining 0.4% represent defective probes.

**Table 1:** **Locus-specific biases on the genomic coverage and error rate of the studied WGA systems using high gDNA input (10 ng)**

	Whole-genome amplification			Genomic coverage ^a^ (%)			Error rate ^b^ (%)		
gDNA extraction	Kit/method	Technology type	gDNA input	All replicates	At least 2/3 replicates	Any replicate	All replicates	At least 2/3 replicates	Any replicate
ChargeSwitch	Non-amplified (Reference)	Non-amplified (Reference)	1.5 μg	97.7%	99.2%	99.6%	00.0%	00.0%	02.0%
ChargeSwitch	REPLI-g	MDA	10 ng	93.8%	98.9%	99.7%	00.6%	01.5%	06.5%
ChargeSwitch	GenomiPhi	MDA	10 ng	89.7%	97.7%	99.5%	00.9%	02.7%	10.7%
ChargeSwitch	Single Cell WGA Kit	QPLS	10 ng	55.6%	68.6%	79.0%	25.9%	34.6%	45.7%
ChargeSwitch	LMA	LMA	10 ng	13.0%	50.2%	94.2%	37.4%	79.0%	96.2%
ChargeSwitch	ExpressLink	LMA	10 ng	23.6%	59.4%	87.0%	43.3%	71.1%	89.9%
ChargeSwitch	LigaFast	LMA	10 ng	19.9%	57.3%	86.3%	44.1%	72.4%	91.8%
ChargeSwitch	Ovation	SPIA	10 ng	66.3%	86.9%	96.9%	06.1%	18.9%	37.3%
Illustra mini Spin Kit	Ovation	SPIA	10 ng	91.8%	96.7%	98.4%	01.6%	03.3%	08.1%

^a^: Genomic coverage or call rates is the proportion of target loci giving positive signals over background over the overall number of loci accounted for on Illumina’s Bovine 50 K SNP Chip. ^b^: Error rate is the proportion of erroneous genotype calls relatively to the non-amplified reference; All replicates (%): % of loci which consistently covered in all performed replicates; At least 2/3 replicates (%): % of loci which consistently covered in At least 2/3 performed replicates; Any replicate (%): % of loci which consistently covered in any performed replicate; LMA: Ligation-Mediated Amplification; MDA: Multiple Displacement Amplification; QPLS: Quasi-random Primed Library Synthesis followed by PCR amplification; SPIA: Single Primer Isothermal Amplification.

**Table 2:** **Locus-specific biases on the genomic coverage and error rate of the studied WGA systems using low gDNA input (15 cells)**

	Whole-genome amplification			Genomic coverage ^a^ (%)			Error rate ^b^ (%)		
gDNA extraction	Kit/method	Technology type	gDNA input	All replicates	At least 2/3 replicates	Any replicate	All replicates	At least 2/3 replicates	Any replicate
ChargeSwitch	Non-amplified (Reference)	Non-amplified (Reference)	1.5 μg	97.7%	99.2%	99.6%	00.0%	00.0%	02.0%
Built-in	REPLI-g	MDA	15 cells	20.9%	73.1%	95.2%	18.4%	42.7%	86.2%
Built-in	GenomiPhi	MDA	15 cells	76.2%	90.7%	98.0%	03.9%	13.6%	26.9%
Built-in	Single Cell WGA Kit	QPLS	15 cells	56.7%	72.0%	83.5%	21.1%	31.3%	44.6%
ChargeSwitch	LMA	LMA	15 cells	07.6%	29.0%	63.8%	62.1%	87.0%	97.8%
ChargeSwitch	ExpressLink	LMA	15 cells	13.7%	43.3%	77.1%	53.0%	80.5%	94.6%
ChargeSwitch	LigaFast	LMA	15 cells	09.1%	32.5%	66.8%	58.0%	84.9%	96.9%
Quick gDNA MicroPrep	Ovation	SPIA	15 cells	11.7%	39.3%	75.1%	44.9%	77.0%	94.8%

^a^: Genomic coverage or call rates is the proportion of target loci giving positive signals over background over the overall number of loci accounted for on Illumina’s Bovine 50 K SNP Chip. ^b^: Error rate is the proportion of erroneous genotype calls relatively to the non-amplified reference; All replicates (%): % of loci which consistently covered in all performed replicates; At least 2/3 replicates (%): % of loci which consistently covered in At least 2/3 performed replicates; Any replicate (%): % of loci which consistently covered in any performed replicate; LMA: Ligation-Mediated Amplification; MDA: Multiple Displacement Amplification; QPLS: Quasi-random Primed Library Synthesis followed by PCR amplification; SPIA: Single Primer Isothermal Amplification.

To determine whether the loci which failed to provide a genotyping call varied randomly between replicates of a method or resulted from a systematic sequence based/locus effect, the number of loci giving positive signal in any, at least 2 or all replicates were compiled (Tables 1 and 2, Additional file 1: Figure S1). We compared these with the expected number of such an occurrence under a binomial process where the errors are randomly distributed amongst loci and the success rate is equal to the mean call rate of the method. No difference between the two measurements was found (P = 1, 95% confidence interval = [−0.02, 0.02]), indicating an absence of locus-specific effect on genotyping call rate. Applying the same test on the global error rates showed a significant difference (P = 8.3 e-6, 95% confidence interval = [−0.16, −0.07]), most likely due to the varying error rates between the different error types (allele drop-out, allele drop-in) which can only occur when the reference genotype is either heterozygous or homozygous, respectively.

### Impact of gDNA output from whole-genome amplification on genotyping performance

**Figure 3:**
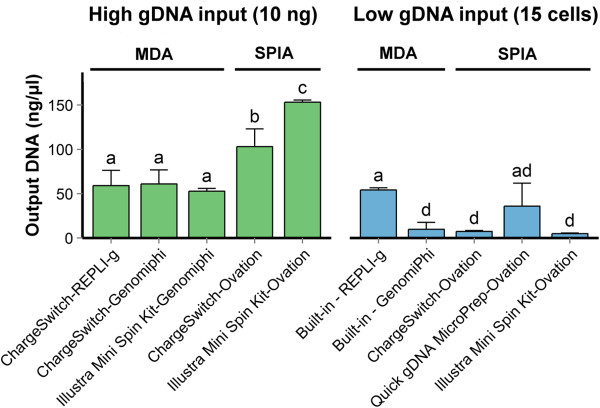
**Yield of amplified gDNA using different combinations of commercial gDNA extraction kits and WGA technologies.** When starting with the recommended minimal input (*i.e.* 10 ng), all kits performed well, however SPIA-based WGA systems produced the most high quantity outputs than the MDA-based WGA systems. Whereas with gDNA obtained from 15 cells, only the REPLI-g Mini kit (MDA-based WGA) and Quick gDNA MicroPrep-Ovation WGA System (SPIA-based WGA) offered the best amplified gDNA output. Error bars represent one standard deviation. Bars with different letters represent data that are significantly different (p<0.05), as determined by Post-hoc analyses using Games-Howell test. MDA: multiple displacement amplification; SPIA: single-primer isothermal amplification.

The DNA yields following WGA were measured for MDA- and SPIA-based WGA technologies (Figure 3). The amount of DNA produced by WGA approaches did not follow the same patterns as genotyping performance across those same methods, ruling it out as a limiting factor. For instance, while the gDNA output for Illustra GenomiPhi V2 DNA amplification kit (MDA-based WGA) was significantly lower (P<0.01) than the REPLI-g Mini Kit (MDA-based WGA), it still produced the most satisfactory genotyping performance.

### Correlation between the genotyping call rate and error rate

**Figure 4:**
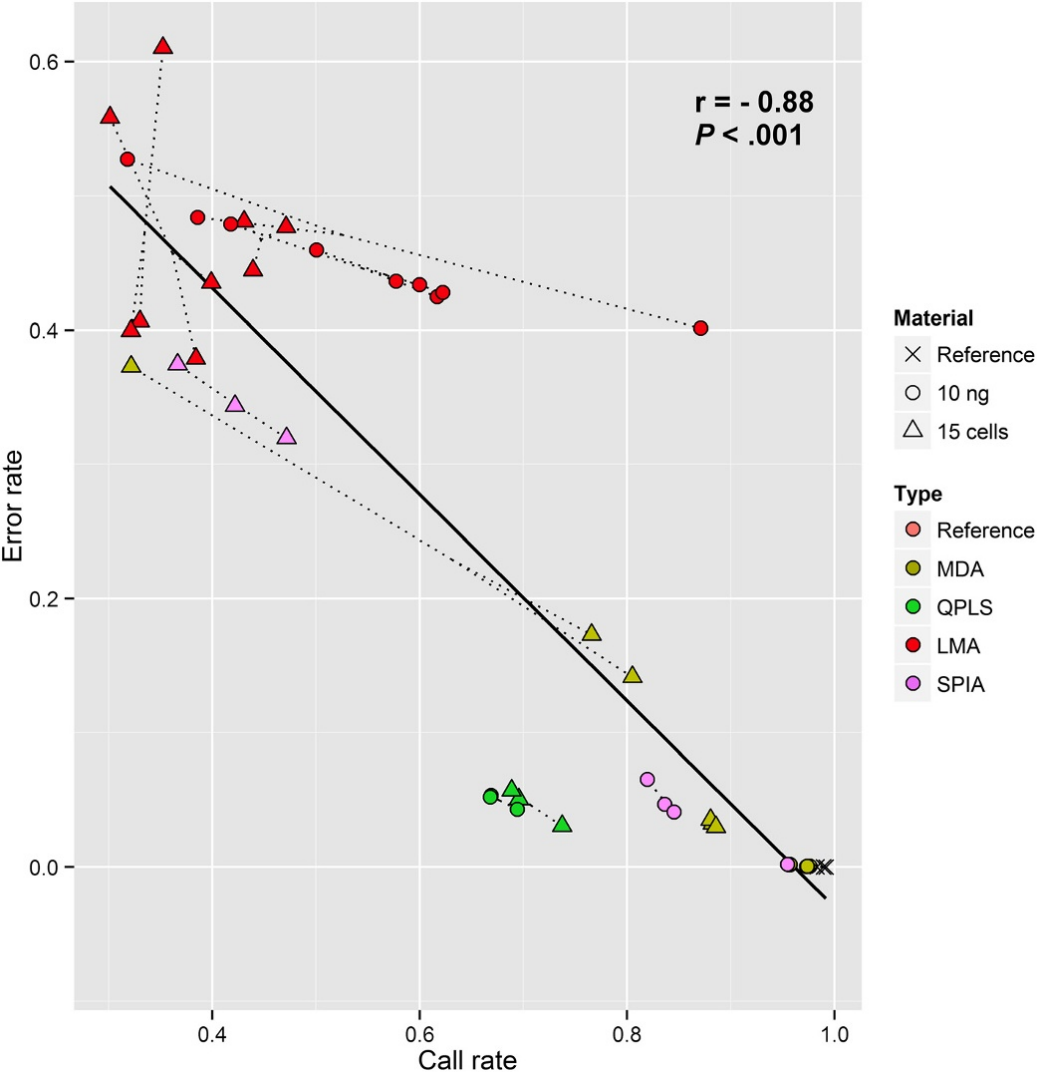
**Correlation between the genotyping call rate and error rate.** Dotted lines join replicates from the same method together. A significant high negative correlation (*r* = −0.88, P<0.001) exists between the genotyping call rate and error rate of the examined WGA technologies, indicating that higher call rates also lead to smaller error rates. Pearson’s product moment correlation coefficient calculated to determine the relationship between the genotyping call rate and error rate. LMA: Ligation-Mediated Amplification; MDA: Multiple Displacement Amplification; QPLS: Quasi-random Primed Library Synthesis followed by PCR amplification; SPIA: Single Primer Isothermal Amplification.

Given that, for example, a loss of gDNA template on an heterozygous locus can lead to either an absence of genotyping call (whenever both alleles are lost) or an allele drop-out (whenever only one allele is lost), it is expected that the genotyping call rates and error rates will be at least partially correlated. A significantly high negative correlation (*r* = − 0.88, P<0.001) was found between the genotyping error rate and call rate of the examined WGA technologies under both types (high and low) of gDNA inputs. This indicates that for any WGA method, a higher call rate will also lead to a much smaller error rate (Figure 4).

### Determining genotyping quality score threshold

**Figure 5:**
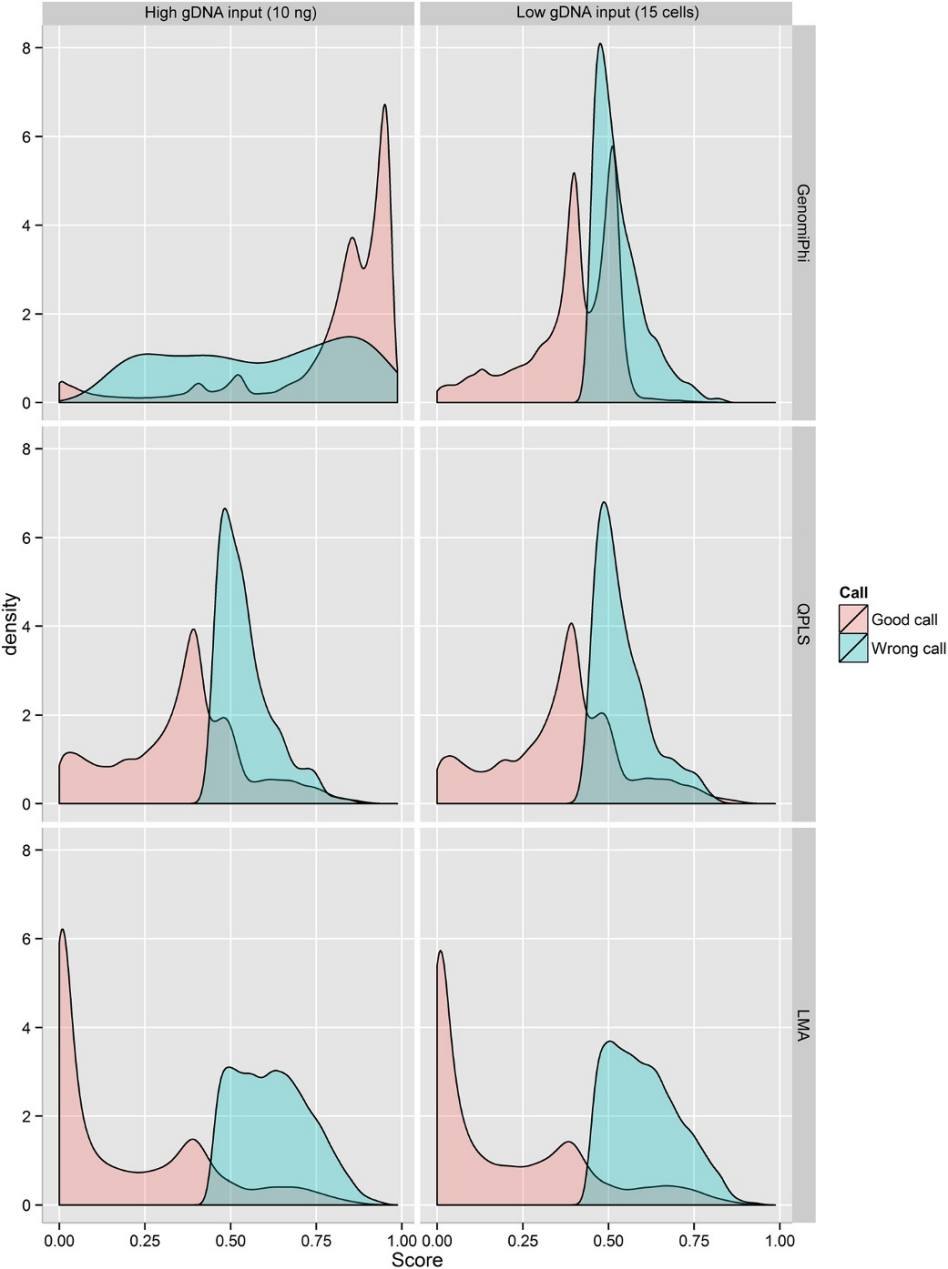
**Density plot quality scores for erroneous and correct genotypes.** The MDA row represents the results obtained from the Illustra GenomiPhi V2 DNA amplification kit (MDA-based WGA). LMA: Ligation-Mediated Amplification; MDA: Multiple Displacement Amplification; QPLS: Quasi-random Primed Library Synthesis followed by PCR amplification; SPIA: Single Primer Isothermal Amplification.

Since the Illumina platform generates a quality score for each locus, we hypothesized that it would be possible to establish a minimal quality cut-off value that would eliminate most of the erroneous genotypes. To test this, the distribution of errors in relation to their call quality score was plotted (Figure 5). Errors were shown to be distributed across the entire range of quality scores, and it was therefore not possible to determine an effective quality score threshold that would eliminate most errors.

### Impact of poor genotype precision on gender determination

The gender of the samples was correctly determined from the genotype information using all four WGA technologies with 10 ng of DNA. However, when the gDNA input was limited to the gDNA obtained from 15 cells, erroneous sexing was observed in the case of the MDA-based WGA (REPLI-g Mini kit) technology (Additional file 2: Table S1). These sex determination errors are attributable to erroneous genotypes.

### Impact of genotyping errors on calculation of genetic merit, the importance of the genotyping reference and imputation

**Figure 6:**
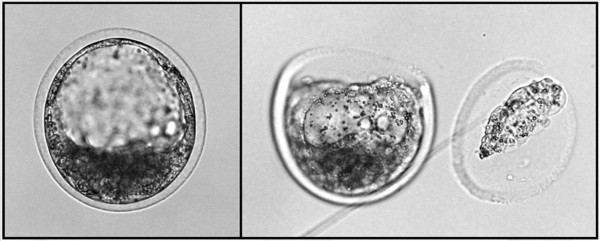
**Embryonic biopsy specimen obtained from trophoblast cells of Day 7.5 bovine embryos.** The removed trophoblast cells provide the gDNA that was subjected to the selected WGA procedure. Following analysis, the genotypic information in conjunction with the genomic imputation were used to estimate bovine genomic breeding value.

To test the effectiveness of genetic merit determination from an embryonic biopsy, the procedure that resulted in the best overall performance was used (Illustra GenomiPhi V2 DNA amplification kit). A total of 226 embryo biopsies were obtained for genotyping (Figure 6). Since genomic coverage was incomplete, the missing genotypes were imputed using the known genotypes of the parents or the common haplotypes found in the Holstein population when parental genotypes were not available. All missing calls were thus filled-in and over 95% of errors were found and corrected, going from a total of 5,286 ± 1,439 errors (12.4 ± 3.4% of total calls) to only 266 ± 188 errors (0.6 ± 0.4% of total calls) in pre- and post-imputation, respectively.

**Figure 7:**
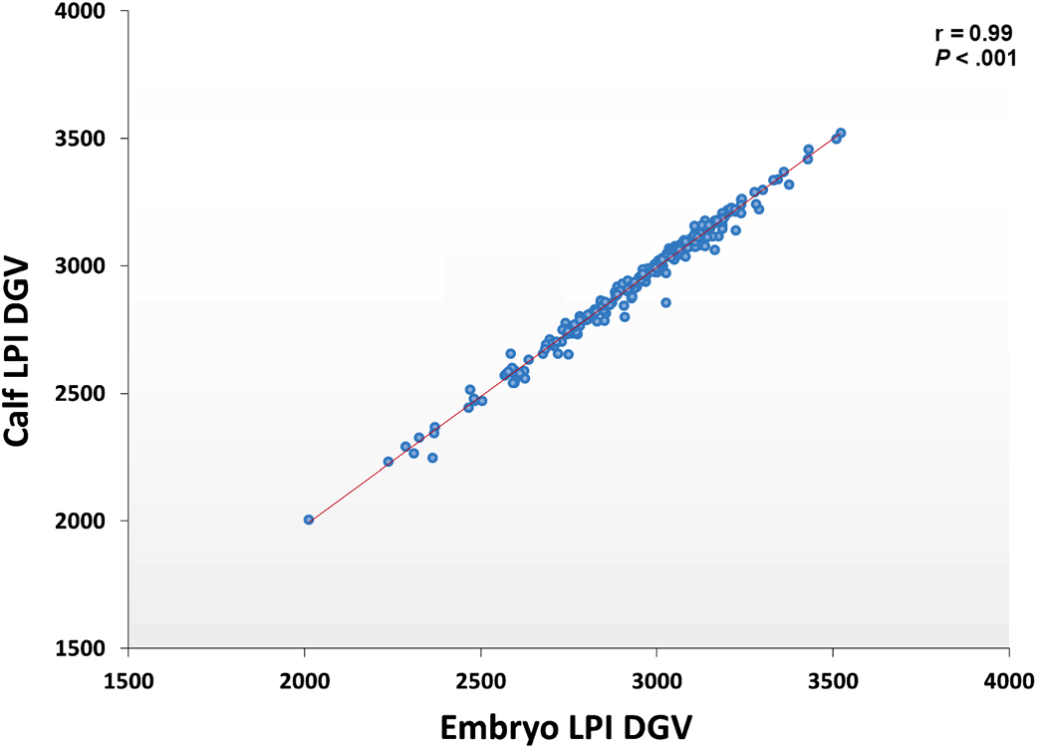
**The correlation of genetic values between the bovine embryo and the corresponding post-natal calf.** The scatter chart showing the divergence between the estimated breeding values based on gDNA obtained from embryonic biopsy (trophoblast, D7.5) and based on the gDNA obtained from corresponding calf tail hair follicles after birth (n = 226). The Pearson correlation coefficient calculated to determine the relationship between the genetic values between the bovine early embryo and the corresponding post-natal calf. DGV: Direct Genomic Values; LPI: Life Profit Index.

Embryos (n = 226) were then transferred and post-natal genotypes were generated from tail hair follicle gDNA collected from the corresponding calves (n = 226), providing sufficient material to make WGA unnecessary. Genetic merit values were calculated both from the imputed WGA-derived embryo genotypes and from the corresponding post-natal genotypes (Figure 7). There was a 99% correlation (P<0.001) between the genetic values calculated from the embryonic biopsy (after genotype imputation) and from the corresponding calf hair follicles. The remaining genotype imprecision resulted in a mean divergence of 106 ± 68 pts of direct genetic value. These minor variations did not have any impact on genetic merit ranking.

## Discussion

The advent of the BovineSNP50 Genotyping BeadChip (Illumina, San Diego, CA, USA) has revolutionized dairy cattle selection [20, 41]. Commercial providers of cattle embryos are now under increasing pressure to determine the genetic makeup of their product. This information increases bovine early embryo value and reduces costs by ensuring the transfer of only high-merit bovine early embryos of the desired gender.

Embryo biopsy is routinely performed for various applications, such as sexing or detection of single-gene defects and coat color. These biopsies remove about 15 cells, representing approximately 10% of the cells present in the trophoblast at the blastocyst stage. However, providing reliable evaluation of genetic merit on a routine basis using only the minute amount of material that can be obtained from an embryo biopsy has been challenging [34–36]. Since a diploid mammalian cell contains approximately 6 pg of genomic DNA, only 90 pg can be obtained from a 15 cells biopsy. The current “minimum” input required by the majority of available commercial kits is generally around 10 ng, a thousand times more. The challenge is therefore to find or adapt a robust genetic analysis system for working with such small amounts of starting material. In the present study, we examined the performance of different PCR-based and non-PCR-based WGA technologies, namely MDA, QPLS, LMA and SPIA, as means of amplifying whole-genomes extracted from very small biopsies for the purpose of calculating the breeding value of pre-transfer bovine embryos.

*In vitro* culture of cells from biopsy has been proposed as a means of increasing the amount of starting material [38]. We examined this approach prior to testing the different WGA options. However, two out of three biopsies (n = 30) failed to grow in culture (data not shown). This failure was likely due to the small number of biopsied cells. Better success was achieved when starting with larger biopsies (20–30 cells), thus obtaining about 1,000 cells in culture, quite sufficient for WGA. However, larger biopsies also significantly compromised embryo viability [38], which is unacceptable from commercial point of view.

In agreement with others, our evaluation of the different amplification procedures determined that MDA-based WGA generated the best results [42–46]. Although, in our study, the Illustra GenomiPhi V2 DNA amplification kit exhibited the best results when starting with low gDNA input while the QPLS-based WGA produced satisfactory results. By contrast, the LMA-based methods proved to be less efficient. This older technology may be suitable for genotyping a restricted panel of selected loci, but amplification from the ends of genomic fragments is definitely more restrictive than random priming. Difficult templates (containing secondary structures, long homopolymers or CG-rich content) cannot be amplified efficiently when initiation of DNA polymerization occurs solely at the ends. Indeed, premature termination of the reaction will not allow the production of a complete template which should contain the priming sequence in order to initiate the next DNA duplication event. In contrast, all methods based on random priming can reinitiate amplification from incomplete templates, thereby providing a more complete genomic coverage.

The process by which a very small sample can be copied in sufficient numbers to allow downstream analysis is composed essentially of two steps, namely extraction of genomic DNA and whole-genomic amplification. The choice of the extraction process must take into consideration the amount of starting material and the number of samples [47]. In this study, we showed that using the same WGA technology (*i.e.* SPIA using high gDNA input) with different extraction methods resulted in different gDNA outputs after amplification as well as different genotyping performance. Successful sample production involves maximizing yield at both steps (gDNA extraction and WGA), since incomplete release of gDNA from the cells necessarily leads to low genomic coverage at the amplification step, even with the most efficient DNA amplification system.

Very low amounts of gDNA as starting material for WGA can lead to randomly biased amplification, which may eventually introduce various genotyping errors [38, 48–50]. As expected and regardless of the applied method, amplifying whole-genome extracted from only 15 cells resulted in less genomic coverage and higher error percentages than when nanograms of gDNA were amplified, which is in agreement with other studies [51, 52]. The most common metrics used to evaluate WGA platforms are the genotyping call rate and allele dropout rates [53]. Although widely used, genomic coverage or the genotyping call rate is proving to be an incomplete genotyping metric as it does not provide information on the introduction of errors. In practice, low but reliable coverage would be preferable to high but erroneous coverage. Evaluation of the rate of allele dropout, in which a known heterozygous genotype becomes homozygous, requires previous knowledge of the actual genotype which is not available in non-experimental samples. In the present study, the use of an unamplified genotypic reference carried out in three replicates provided the basis for the precise calculation of error rates. Using gDNA recovered from samples containing 15 cells resulted in an overall increase in allele dropout as well as allele drop-in, in which a known homozygous genotype unexpectedly generates a heterozygous call.

One possible explanation for the apparent creation of new alleles could be the mis-attribution of the detected signals by the analysis software due to a high “signal-to-noise” ratio. It is important to note that for platforms such as the Illumina BeadChip the clustering step applied prior to genotype calling can have a large impact on the generated genotypes (data not shown). Compared to unamplified material, gDNA subjected to whole genome amplification will have a larger dynamic range of signal as well as an abundance of missing loci. This makes clusters generated using the former type of samples sub-optimal when calling genotypes with samples of the latter type, as signal that should be rightfully considered as “noise” for amplified material falls within the acceptable calling thresholds for unamplified DNA. The occurrence of genotypes reversion from one homozygous state to another as observed in the present study represents an extreme manifestation of such errors. This could explain the abundance of genotyping errors in all low gDNA input samples, regardless of the applied WGA technology. However, by generating appropriate WGA-specific cluster files it would be possible to optimize detection thresholds and improve call rates. It would require a large number of samples (100 – 1,000) and this template could be used for all subsequent samples. The number of replicates in this study does not allow for these optimizations. As such, all WGA approaches were tested using basic parameters. Other parameters could have benefited other WGA methodologies. Overall, lowering the input DNA negatively affected all WGA by increasing the signal-to-noise ratio.

Another line of evidence for the high signal-to-noise ratio origin for most erroneously called loci is the negative correlation observable between the genotyping call rate and the error rate of a particular method. In contrast with most other microarray methods in which an “absent” measure is the result of intensities falling below a background threshold, in the context of the Illumina BeadChip platform “no call” indicates that the intensities fell outside the bounds of the clustering regions defined for AA, AB and BB genotypes, even if those intensities were high. Noisier data will result in a more random distribution of points, which will both cause points to fall outside of any bounded region (generating “no calls”) or within a random region (creating allele drop-out, but also allele drop-ins).

Variable genomic coverage and erroneous identification of genotypes will have different impact depending on the targeted application. When the goal is to detect precise point mutations in specific target loci, genomic coverage is not a factor as long as the target loci are covered, while genotype precision is crucial. However, for detection of large chromosome defects, genomic coverage is important while precision may be less so, since the high number of data points may compensate for a lack of precision. In the case of sex determination, genotype precision is not crucial, since the test essentially involves detecting the presence of the Y chromosome. However, the SNP panel of the Illumina Bovine 50 K SNP Chip does not contain specific Y chromosome loci. Sex determination is performed by comparing the average proportion of heterozygous genotypes on autosomal chromosomes to that on the X chromosome. Male embryos should not have heterozygous genotypes on the X chromosome. Using this method, sex determination errors will occur more frequently when the allele dropout rate is high, since this reduces the overall number of heterozygous loci. It was therefore not surprising that the REPLI-g Mini kit (MDA-based WGA), which showed the highest dropout for low gDNA input, consistently failed to identify embryo gender.

Based on these comparisons, the selected WGA platform was then tested using embryo biopsies, which may differ in cell content and gDNA quality compared to the standardized *in vitro* culture samples containing 15 cells each. Precision was measured by comparing bovine embryo whole genotypes to corresponding post-natal calf genotypes, the latter data obtained from unamplified gDNA. Since the information obtained from the biopsies was incomplete, genotype imputation was applied to fill-in the gaps using embryo sire and dam data (when available) or data from the general population of genotyped individuals. Genotype imputation based on parental genotypes or genotypes from the general population is already in usage when lower density SNP panels are used [54, 55]. Although data imputation completes the dataset, the presence of erroneous genotypes poses a greater challenge since they are not identifiable in the absence of reference genotypes. The imputation algorithm used in the present study was designed not only to fill-in the missing calls but also to detect and correct most of the errors based on parent information. The remaining errors (266 ± 188 loci) led to a correlation of 0.99 between the genetic merit of the embryo and that of the corresponding calf.

## Conclusion

Not all WGA platforms exhibit equal performance especially with low gDNA input. The very small quantity of input gDNA obtained from embryo biopsies challenges the limits of all current technologies. Even the best-performing WGA platform generated incomplete and erroneous genomic information. These errors cannot be neglected since their sum is sufficient to translate into imprecise genetic evaluations. Genotype imputation, including the correction of genotype errors based on information from parents, is effective and necessary for palliating the limitations of WGA. In our experiments, the average difference between the genetic merit of the bovine embryo and of the corresponding calf was 106 ± 68 pts of LPI, which is only about one seventh of the population averaged standard deviation for LPI. This confirms that this method is robust and provides precise information.

## Methods

### Ethics statement

All animals used in this study were handled following the guidelines provided by the Canadian Council on Animal Care. These guidelines are strictly followed by both the local abattoir and L’Alliance Boviteq who provided all tissues and samples. The study did not require handling animals on university premises.

### Bovine fetal fibroblast culture and cell sorting

Bovine fibroblasts were obtained from leg biopsies of three female Holstein fetuses (≤2 months of gestation) collected at a local slaughterhouse. Minced tissue was incubated in 0.1% trypsin/EDTA for 30 minutes. The cells were washed and incubated at 38.5°C in a 5% CO_2_ atmosphere in 25 cm^2^ flat culture flasks containing DMEM medium (Invitrogen, Burlington, ON, Canada) supplemented with 10% fetal bovine serum and antibiotics. Passages were performed regularly when cells approached confluence. Upon the first passage, an aliquot was taken for gender confirmation by PCR. One culture was kept for downstream analyses. Transferred cells were collected, washed and two 1 μl aliquots were used to obtain approximate counts using a hemocytometer. The remaining cells were divided into samples containing about 70,000 each (*i.e.* about 420 ng of genomic DNA) for preliminary gDNA extraction. In this study, the genomic DNA from 15 cells was considered as “low gDNA input” representing the gDNA routinely obtained for the purpose of embryo biopsy. Therefore to collect the samples containing 15 cells for further analysis, the cells were washed in PBS at the third and sixth passages during cell culture and about 25% of them were sorted and distributed into 96-well plates at exactly 15 cells per well using a fluorescent-activated cell sorter (FACS) (BD SORP FACSAria II; Becton Dickinson, San Jose, CA, USA). To ensure FACS accuracy to seed exactly 15 cells/well, the number of cells per well was visually confirmed in five randomly chosen wells using Trypan Blue and a stereomicroscope. The remaining cells were used for extraction of larger amounts of gDNA using the various commercial systems. In contrast with the “low gDNA input”, which was obtained from 15 cells, the 10 ng gDNA samples were considered as “high gDNA input” and represented the minimal gDNA input for most WGA kits. Aliquots containing this amount were prepared, based on UV absorbance measurement using a NanoDrop ND-1000 spectrophotometer (NanoDrop Technologies Inc., Wilmington, DE, USA). In addition, for both low and high gDNA inputs, three samples containing 1.5 μg of gDNA were prepared using the ChargeSwitch gDNA Micro Tissue kit (#CS11203, Invitrogen, Carlsbad, CA, USA) and set aside to generate the unamplified reference. All gDNA samples were stored at −80°C until use.

### Testing the genomic DNA extraction procedures

Genomic amplification is composed of two main steps, namely gDNA extraction and DNA amplification. These steps are somewhat independent, since different extraction protocols can be fitted with a given amplification platform. Furthermore, gDNA extraction methods differ in terms of their efficiency based on the starting materials and perform more efficiently with higher gDNA input. Therefore, to investigate the most efficient gDNA extraction methods for “low gDNA input”, the efficiency of the extraction systems was initially tested initially with large samples (70,000 cells). To do that, the efficiency of several commonly available commercial gDNA extraction kits, as well as non-commercials (homemade DNA extraction) approaches were investigated. Four column-based commercial kits comprising: (i) DNeasy Blood & Tissue Kit kit (Qiagen, Mississauga, ON, Canada), (ii) QIAamp DNA mini kit (Qiagen, Mississauga, ON, Canada) (iii), Illustra Tissue and Cells genomicPrep Mini Spin Kit (#28-9042-75, GE Healthcare Bio-Sciences Inc., QC, Canada) and (iv) Quick gDNA MicroPrep kit (Zymo Research, Irvine, CA, USA) and a magnetic bead-based commercial kit, namely ChargeSwitch gDNA Micro Tissue kit (Invitrogen, Carlsbad, CA, USA) were tested. All extraction procedures were performed exactly as described in the manufacturers’ instructions. In addition, the examined homemade approaches in this study were one single-step method using proteinase K and a two-steps method using proteinase K treatment followed by phenol extraction. Briefly, samples were incubated overnight at 56°C in 1 mL of SLB buffer (10 mM Tris pH 7.5, 10 mM EDTA, 50 mM NaCl and 0.2% SDS) with proteinase K (100 μg), followed by treatment with RNAse A (100 μg) for 1 h at 37°C, then protein extraction using phenol, phenol:chloroform:isoamyl alcohol (25:24:1) and chloroform:isoamyl alcohol (24:1). Genomic DNA was precipitated with 1/12.5 volume of 3 M NaCl and 2.5 volumes of ethanol (95–100%) and then re-suspended in TE buffer (Sigma, St. Louis, MS, USA). All extractions were assessed in parallel and performed in triplicate, and gDNA recovery was evaluated using UV absorbance (420 ng corresponding to total recovery from 70,000 cells). The integrity of the recovered gDNA was assessed using a DNA size marker (Invitrogen 1 kb Plus, Burlington, ON, Canada) on a 1% agarose gel stained with ethidium bromide.

### Producing the genotype reference

Unamplified samples containing 1.5 μg of gDNA were analyzed for single-nucleotide polymorphisms (SNP) using an Illumina Bovine 50 K Chip microarray (Illumina, San Diego, CA, USA). Three replicates of each sample were thus analyzed to provide the reference genotypes against which the genotypes generated from the high and low gDNA input WGA procedures were compared. Since the cell samples were collected from two distinct culture transfers, the reference was generated both times to ensure that culturing did not modify the karyotype. When genotypes between the reference duplicates differed, the consensus genotype was used. If no consensus genotype could be determined, a “no call” was generated.

### Whole-genome amplification (WGA)

Whole-genome amplification (WGA) was performed using commercial kits based on MDA, QPLS and SPIA technologies as well as ligation-mediated PCR amplification (LMA). The procedures for all of the commercial kit were performed according to the manufacturers’ recommended protocols. Two widely used MDA-based WGA systems were tested, namely the Illustra GenomiPhi V2 DNA amplification kit (#25-6600-30, GE Healthcare Bio-Sciences Corp., QC, Canada) and the REPLI-g Mini Kit (#150023, Qiagen, Mississauga, ON, Canada). The QPLS technology is the principle of the Single Cell WGA Kit (#E2620, New England Biolab, Pickering, ON, Canada). The Ovation WGA System (#6100-12, NuGEN, San Carlos, CA, USA) was chosen as the commercially available SPIA-based WGA kit. The general ligation-mediated amplification (LMA) procedure as described previously by Klein *et al.* [56] was used with some modifications. To optimize the protocol, different ligases including LigaFast Rapid DNA Ligation System (#M8221, Promega, Madison, WI, USA) and ExpressLink T4 DNA Ligase (#A13726, Invitrogen, Carlsbad, CA, USA) were tested. All of the LMA-amplified samples were purified using the QIAquick PCR purification kit (Qiagen, Chatsworth, CA, USA) prior to amplification. Each technology was tested using three replicates. Following completion of the reaction, 5 μl were run on agarose gel to confirm successful amplification, and the remaining material was kept at −20°C until processed for genotyping.

### Evaluation of WGA quality using a single-nucleotide polymorphism microarray

Genotyping was conducted using the Illumina Bovine SNP50 BeadChip (Illumina, San Diego, CA, USA) on the platform of a commercial service provider (DNA Landmark, St-Jean-sur-Richelieu, QC, Canada) with the Illumina Genome Studio software. Amplified gDNA was standardized at 250 ng for analysis. Genomic coverage and error rates were estimated for all samples. Genomic coverage was defined as the number of loci for which the sample provided a genotype call, regardless of whether the call was correct. The rates for the various types of genotyping error were calculated by comparing the genotypes from the WGA samples to the reference produced from unamplified samples (described above). All conditions (reference and different WGA technologies) were tested in triplicate to provide an indication of data repeatability (variability).

### Evaluation of the impact of gDNA input on performance of different WGA technologies

To assess the reliability of the tested WGA technologies under high and low gDNA input, call rate, overall error rate (comprising homozygosity reversal, heterozygosity gain and allele drop-out) and finally the proportion of correct calls were measured and statistically analysed. One-way ANOVA with Games-Howell post-hoc test was performed, due to the apparent heteroscedasticity of the data. To determine if any locus-specific effect could be detected, the number of loci where any, at least 2 or all replicates provided a call was computed. The same analysis was then performed for loci that provided an erroneous call. Using a paired t-test, those were compared with the expected number of such occurrences under the assumption of a binomial process where the errors are randomly distributed amongst loci and the success rate is equal to the mean call or error rate of the method. Finally, the correlation between the measured genotyping error rate (including all types of errors) and the genotyping call rate were calculated using Pearson’s product moment correlation coefficient.

### *In vivo*bovine blastocyst production and embryo biopsy

On Day 8–12 post-estrus, follicles with a diameter larger than 8 mm from Holstein heifers and cows were eliminated by ultrasound-guided transvaginal aspiration. Thirty-six hours later, administration of FSH (Folltropin-V, Bioniche Animal Health, Bellville, ON, Canada) was started. A total of eight injections were given (two per day) for a total of 400 mg of FSH, in doses decreasing from 60 mg to 20 mg. A 500-μg dose of prostaglandin F2α analogue (Estrumate, Intervet, Kirkland, QC, Canada) was administered with each of the two final FSH injections to trigger luteolysis. The animals exhibited estrus about 36 h after the final FSH/Estrumate injection and were inseminated twice, 12 and 24 h post-estrus with the pooled semen used for *in vitro* fertilization. Bovine embryos at blastocyst stage were recovered by uterine flushing 7.5 days (D7.5) after the first insemination. Expanded blastocyst of good quality (IETS quality grades 1 and 2) were washed and about 15 trophoblast cells were biopsied using a micro-blade (Bioniche, Pulman Washington, USA) mounted on a Leitz micromanipulator (Leitz, Grand Rapids, MI, USA). Genomic DNA extraction and WGA were carried out immediately after biopsy in order to maximize the quality of the results.

### Embryo transfer and post-natal sample collection

In order to evaluate the efficiency of the selected WGA platform in a commercial embryo production setting, embryos were transferred after the biopsy procedure to suitably prepared recipient cows. For the purpose of this study, only biopsied embryos (n = 226) which resulted in successful calving following embryo transfer were considered. Unamplified gDNA from tail hair follicles of the corresponding live calves was sent to the commercial service provider for genotyping (DNA Landmark, St-Jean-sur-Richelieu, QC, Canada) on the Illumina Bovine SNP50 BeadChip platform. The accuracy of the WGA-derived genotyping was determined by comparing the genotypes of bovine early embryo and its corresponding calf.

### Using embryo genotypes to calculate estimated breeding values

Since even the most efficient WGA platform could not provide complete genomic coverage, genotype imputation of the SNP data obtained from the Illumina platform is necessary in order to complete the genotypic information required for precise estimation of breeding value. The genotypes of the parents are usually based on biological samples from live animals which contain enough gDNA for pre-amplification to be unnecessary. Therefore, these genotypes tend to be highly accurate and the corresponding information can be used to correct some of the genotyping errors in the embryo biopsy that are found whenever Mendelian inconsistencies are detected. The proportion of corrected or removed genotypes can then be used as an indicator of embryo genotype quality. After this a genotype imputation program can be used to fill-in missing genotypes. In this study, imputation was carried out using a modified version of FImpute V2.2 [57], which has special features for embryo genotyping. This software performs a combined family and population genotype imputation. The quality of the resulting genotypes was assessed through various criteria such as final missing rate, homozygosity rate, and divergence between traditional and genomic inbreeding. The bovine embryo genotypes obtained after imputation were used to calculate direct genomic values (DGV), which reflect the effects of 50 k markers in official genomic evaluations calculated by the Canadian Dairy Network (CDN) for each evaluated trait. These DGV were combined across traits to calculate the DGV for the lifetime profit index (LPI), the national selection index. The DGV obtained from the genotypes of embryo biopsies was then compared to the DGV obtained from the genotypes of the corresponding live calves, those which resulted from successful pregnancies following embryo transfer. The 50 k genotypes of live calves were obtained from unamplified hair follicle DNA, which is the most common practice in dairy industry. This comparison between DGV is particularly important in terms of assessing the applicability of embryo genotyping to the dairy industry, since the DGV of each animal is the main criterion used to select each animal, in combination with its parent average (but the parent average is not affected by the animal’s genotype).

### Statistical analysis

One-way ANOVA followed by Games-Howell post-hoc test was used as the between groups statistical analysis for both high and low gDNA inputs. A Pearson’s product moment correlation coefficient procedure was used to identify the correlation between genotyping error rate and call rate as well as the imputed WGA-derived embryo genotypes and from the post-natal genotype. The P<0.05 was considered significant. Data were reported as mean ± SD.

## Electronic supplementary material

Additional file 1: Figure S1: Comparison of the three replicates in terms of the numbers of loci that provided positive genotype calls, for each tested WGA technologies under high and low gDNA input**.** For high gDNA input (10 ng), MDA-based WGA kits showed consistently the highest reproducibility. SPIA-based technology showed very high reproducibility in conjunction with the Illustra MiniSpin kit but not with ChargeSwitch gDNA Micro Tissue kit. Results obtained using LMA-based methods were the least reproducible regardless of the type of DNA polymerase. For low gDNA input (15 cells), the highest reproducibility was achieved for Illustra GenomiPhi V2 DNA amplification kit (MDA-based WGA) followed by the Single Cell WGA Kit (QPLS-based. **LMA:** Ligation-Mediated Amplification; **MDA:** Multiple Displacement Amplification; **QPLS:** Quasi-random Primed Library Synthesis followed by PCR amplification; **SPIA**: Single Primer Isothermal Amplification. (PDF 453 KB)

Additional file 2: Table S1: Sex determination from the genotyping results. **F:** Female; **LMA:** Ligation-Mediated Amplification; **M:** Male; **MDA:** Multiple Displacement Amplification; **QPLS:** Quasi-random Primed Library Synthesis followed by PCR amplification; **SPIA:** Single Primer Isothermal Amplification. (PDF 191 KB)

## Abbreviations

CDN: Canadian dairy network
DGV: Direct genomic values
ET: Embryo transfer
LMA: Ligation-mediated amplification
LPI: Life profit index
MDA: Multiple displacement amplification
QPLS: Quasi-random primed library synthesis followed by PCR amplification
SLB: Spheroid lysis buffer
SNP: Single nucleotide polymorphism
SPIA: Single primer isothermal amplification
WGA: Whole-genome amplification.
