# Supplementary material for: Impact of whole-genome amplification on the reliability of pre-transfer cattle embryo breeding value estimates
Source: BMC Genomics. 2014 Oct 12;15(1):889. doi: 10.1186/1471-2164-15-889 (PMC4201692; doi:10.1186/1471-2164-15-889)
Supplement: Supplementary file 1 — Additional file 1: Figure S1: Comparison of the three replicates in terms of the numbers of loci that provided positive genotype calls, for each tested WGA technologies under high and low gDNA input. For high gDNA input (10 ng), MDA-based WGA kits showed consistently the highest reproducibility. SPIA-based technology showed very high reproducibility in conjunction with the Illustra MiniSpin kit but not with ChargeSwitch gDNA Micro Tissue kit. Results obtained using LMA-based methods were the least reproducible regardless of the type of DNA polymerase. For low gDNA input (15 cells), the highest reproducibility was achieved for Illustra GenomiPhi V2 DNA amplification kit (MDA-based WGA) followed by the Single Cell WGA Kit (QPLS-based. LMA: Ligation-Mediated Amplification; MDA: Multiple Displacement Amplification; QPLS: Quasi-random Primed Library Synthesis followed by PCR amplification; SPIA: Single Primer Isothermal Amplification. (PDF 453 KB) [file 12864_2014_6558_MOESM1_ESM.pdf]

Supplementary figures

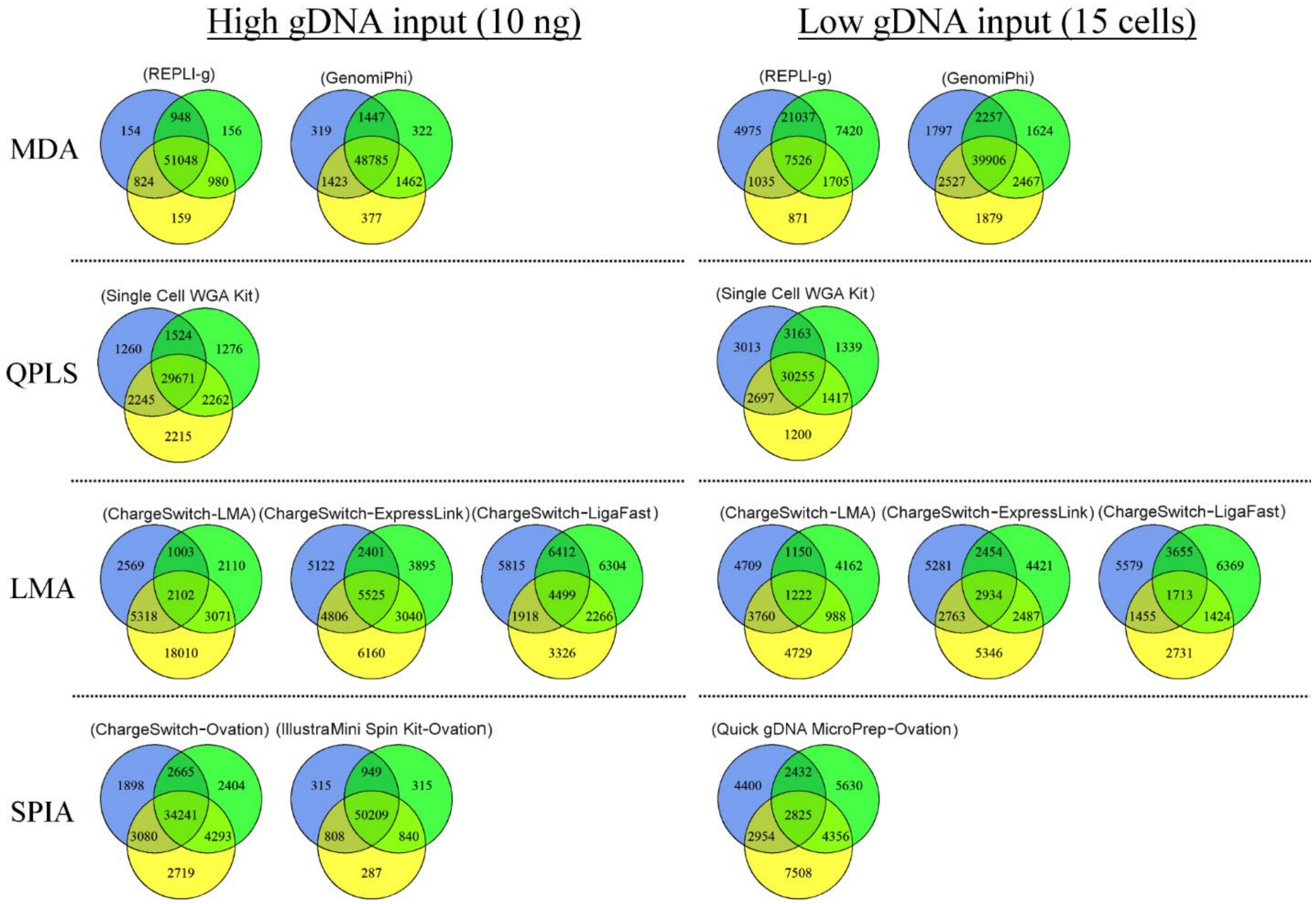

Figure S1. Comparison of the three replicates in terms of the numbers of loci that provided positive genotype calls, for each tested WGA technologies under high and low gDNA input. For high gDNA input (10 ng), MDA-based WGA kits showed consistently the highest reproducibility. SPIA-based technology showed very high reproducibility in conjunction with the Illustra MiniSpin kit but not with ChargeSwitch gDNA Micro Tissue kit. Results obtained using LMA-based methods were the least reproducible regardless of the type of DNA polymerase. For low gDNA input (15 cells), the highest reproducibility was achieved for Illustra GenomiPhi V2 DNA amplification kit (MDA-based WGA) followed by the Single Cell WGA Kit (QPLS-based. **LMA:** Ligation-Mediated Amplification**; MDA**: Multiple Displacement Amplification; **QPLS:** Quasi-random Primed Library Synthesis followed by PCR amplification**; SPIA**: Single Primer Isothermal Amplification.
