# Supplementary material for: Impact of whole-genome amplification on the reliability of pre-transfer cattle embryo breeding value estimates
Source: BMC Genomics. 2014 Oct 12;15(1):889. doi: 10.1186/1471-2164-15-889 (PMC4201692; doi:10.1186/1471-2164-15-889)
Supplement: Supplementary file 2 — Additional file 2: Table S1: Sex determination from the genotyping results. F: Female; LMA: Ligation-Mediated Amplification; M: Male; MDA: Multiple Displacement Amplification; QPLS: Quasi-random Primed Library Synthesis followed by PCR amplification; SPIA: Single Primer Isothermal Amplification. (PDF 191 KB) [file 12864_2014_6558_MOESM2_ESM.pdf]

Supplementary Tables

Table S1. Sex determination from the genotyping results.

	gDNA extraction	Whole-genome amplification		Replicate		
		Kit/method	Technology type	#1	#2	#3
10 ng of gDNA	ChargeSwitch	REPLI-g	MDA	F	F	F
	ChargeSwitch	GenomiPhi	MDA	F	F	F
	ChargeSwitch	Single Cell WGA Kit	QPLS	F	F	F
	ChargeSwitch	LMA	LMA	F	F	F
	ChargeSwitch	ExpressLink	LMA	F	F	F
	ChargeSwitch	LigaFast	LMA	F	F	F
	ChargeSwitch	Ovation	SPIA	F	F	F
	Illustra Mini Spin Kit	Ovation	SPIA	F	F	F
15 cells	Built-in	REPLI-g	MDA	M	M	M
	Built-in	GenomiPhi	MDA	F	F	F
	Built-in	Single Cell WGA Kit	QPLS	F	F	F
	ChargeSwitch	LMA	LMA	F	F	F
	ChargeSwitch	ExpressLink	LMA	F	F	F
	ChargeSwitch	LigaFast	LMA	F	F	F
	Quick gDNA MicroPrep	Ovation	SPIA	F	F	F

F: Female; **LMA:** Ligation-Mediated Amplification**; M:** Male; **MDA**: Multiple Displacement Amplification; **QPLS:** Quasi-random Primed Library Synthesis followed by PCR amplification**; SPIA**: Single Primer Isothermal Amplification.
